# Association of soy intake and cooking methods with colorectal polyp and adenoma prevalence: findings from the extended Lanxi pre-colorectal cancer cohort (LP3C)

**DOI:** 10.3389/fnut.2024.1390143

**Published:** 2024-06-19

**Authors:** Weifang Zheng, Xunan Lin, Meng Zhu, Hao Ye, Xiaodong Hu, Xiaohui Liu, Lixiang Hu, Youyou Zheng, Peiling Hu, Pan Zhuang, Jingjing Jiao, Yu Zhang

**Affiliations:** ^1^Lanxi Hospital of Traditional Chinese Medicine, Jinhua, Zhejiang, China; ^2^Department of Nutrition, School of Public Health, Zhejiang University School of Medicine, Zhejiang, Hangzhou, China; ^3^School of Basic Medicine, Zhejiang Chinese Medical University, Zhejiang, Hangzhou, China; ^4^Lanxi Red Cross Hospital, Jinhua, Zhejiang, China; ^5^Department of Food Science and Nutrition, Zhejiang Key Laboratory for Agro-Food Processing, College of Biosystems Engineering and Food Science, Zhejiang University, Zhejiang, Hangzhou, China

**Keywords:** colorectal polyps, adenoma, soy products, cooking methods, Lanxi precolorectal cancer cohort, polyp subtypes

## Abstract

**Background:**

Limited research has explored the association between dietary soy products and colorectal polyps and adenomas, with insufficient attention given to cooking methods and subtypes of polyps. This study aimed to comprehensively assess the relationship between soy intake, its cooking methods, and the risk of colorectal polyps and adenomas within a high-incidence population of colorectal cancer (CRC) in China.

**Methods:**

Data were derived from 14,903 participants aged 40–80 years, enrolled in the extended Lanxi Pre-colorectal Cancer Cohort (LP3C) between March 2018 and December 2022. This cross-sectional study is based on the participants’ baseline information. Long-term dietary information was collected through a validated food frequency questionnaire (FFQ), and colorectal polyps and adenomas were identified through electronic colonoscopy. Employing multivariate logistic regression, results were expressed as odds ratios (ORs) with their corresponding 95% confidence intervals (95% CIs).

**Results:**

4,942 cases of colorectal polyps and 2,678 cases of adenomas were ascertained. A significant positive association was found between total soy intake and the occurrence of polyps/adenomas. Considering cooking methods, a notable increase in polyp risk was associated with the consumption of fried soys while no association was detected for boiled or marinated soys. Furthermore, total soy intake demonstrated associations with large and multiple polyps, polyps Yamade-typed less than II, and polyps across all anatomical subsites.

**Conclusion:**

Within the high-risk CRC population in China, increased soy product intake was linked to a higher risk of polyps, primarily attributed to the consumption of fried soys. This suggests that modifying cooking methods to avoid fried soys may serve as a preventive strategy for colorectal polyps.

## 1 Introduction

The GLOBOCAN 2020 database reveals alarming global statistics for colorectal cancer (CRC), with approximately 2 million new cases and nearly 1 million deaths reported in 2020. In China, during the same period, deaths constituted about 30% of the global total, and the age-standardized mortality rate (ASMR) remained notably high worldwide (1). The escalating incidence and mortality rates of CRC in China (2, 3), attributed to factors such as the Westernization of lifestyle and dietary patterns, underscore the substantial burden posed by this disease (4). Colorectal polyps and adenomas, recognized as precursor lesions of CRC and associated with a heightened risk of malignancy, further contribute to the gravity of the situation (5, 6). Given the high preventability of CRC through dietary modifications (7), a growing body of evidence establishes the link between dietary factors and the risk of colorectal polyps and adenomas (8–11). Specifically, dietary fiber emerges as a potential mitigating factor in CRC incidence (12).

Legumes, characterized by their richness in protein, dietary fiber, micro-nutrients, and phytochemicals such as isoflavones, constitute commonplace items in daily diets (13), especially soy. Despite their nutritional significance, limited research has delved into the relationship between legume consumption and CRC risk, leaving the nature of this association unclear (11, 14–17). Additionally, due to the multifaceted culinary culture in China where a plethora of cooking methods are commonly employed, these food processing methods exert diverse impacts on the composition of foods and nutrients (18–20). Hence, different types of cooking methods should be considered when evaluating dietary intake among Chinese population. Cooking methods that entail the use of high temperatures and significant quantities of oil, particularly deep frying, have been correlated with an increased incidence of CRC (20, 21). Moreover, a rise in the consumption of dietary trans fatty acids has been implicated in increasing the risk of CRC (22).

In light of these considerations and China’s unique dietary characteristics, we conducted this study based on the Lanxi Pre-colorectal Cancer Cohort (LP3C) to investigate the relationship between soy intake under different cooking methods and the risk of colorectal polyps and adenomas among high-risk individuals in China. The primary objective is to fill research gaps and provide valuable evidence from a large population, enabling the formulation of practical dietary recommendations for the daily prevention of CRC.

## 2 Materials and methods

### 2.1 Participants

The LP3C study, situated in Lanxi, Zhejiang Province, a recognized high-incidence area for CRC, aimed to implement a 5-year CRC screening program for 500,000 residents aged 40 to 80 years, residing in Jinhua for at least 6 months. Commencing recruitment in March 2018, high-risk CRC residents were identified from 6 subdistricts, 7 towns, and 3 villages. High risk for CRC was defined by specific conditions: (1) personal history of colorectal polyps or CRC, (2) family history of CRC, or (3) a positive fecal occult blood test. Participants meeting any of these criteria were classified as high risk for CRC (23). A comprehensive overview of the study design and methods has been previously published (24). Briefly, face-to-face interviews facilitated the collection of participant information, encompassing demographic characteristics, socioeconomic status, diet, lifestyle factors, and personal and family medical history. Subsequently, participants underwent colonoscopy within 1 month at the Lanxi Red Cross Hospital. The LP3C study has now completed the first five-year screening and thus the current study is an extended analysis that utilized data from 2018 to December 2022.

**Figure 1:**
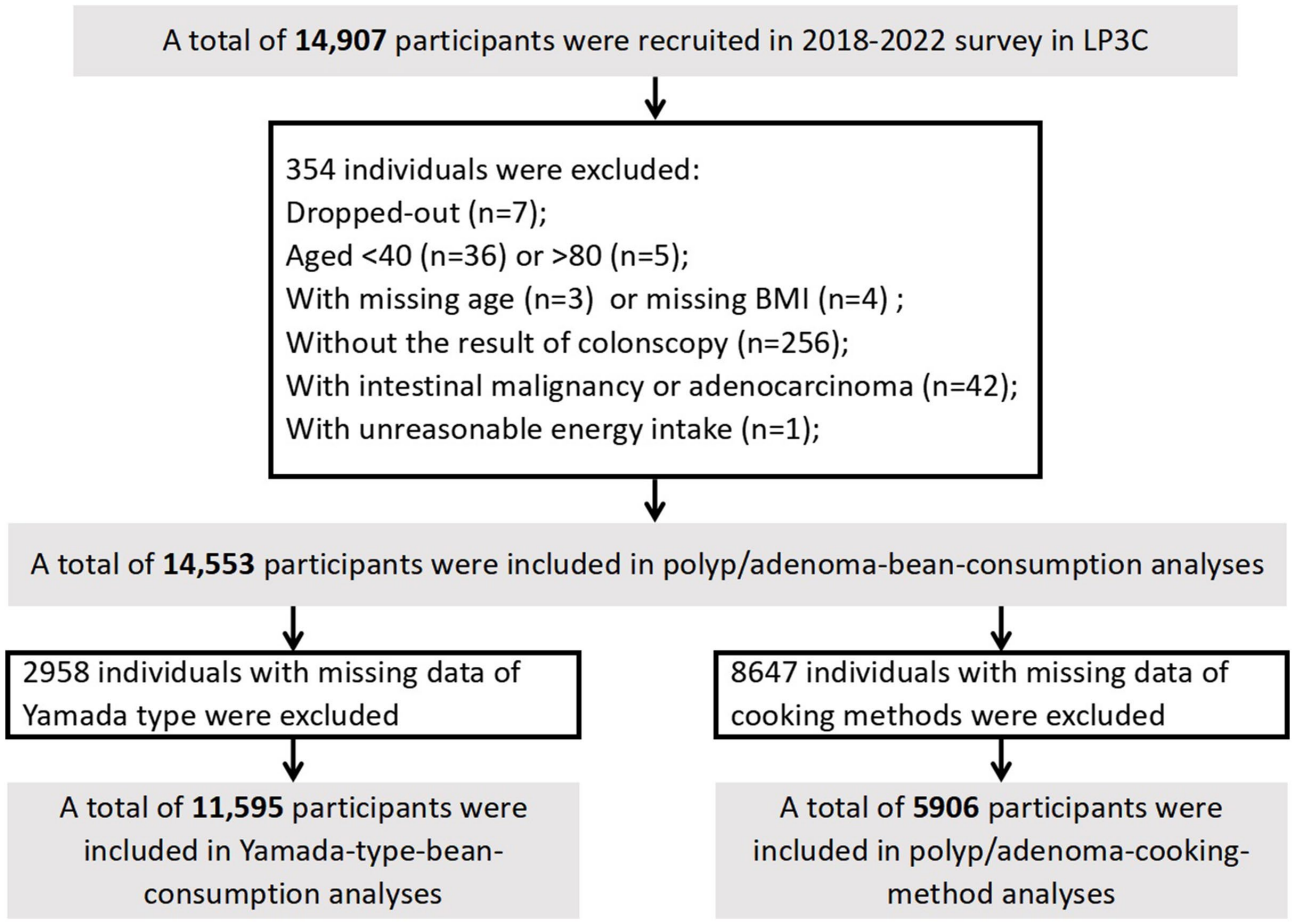
Flow chart of study participants in current the Lanxi Pre-Colorectal Cancer Cohort (LP3C) study.

A total of 14,907 participants were enrolled during 2018 to 2022. We conducted an initial cross-sectional study with the baseline data of participants to investigate the impact of soy products and cooking methods on colorectal polyps/adenomas. Exclusions were made for dropouts (*n* = 7), age < 40 or > 80 (*n* = 41), missing age (*n* = 3), missing body mass index (BMI; *n* = 4), missing colonoscopy results (*n* = 256), individuals with intestinal malignancy or adenocarcinoma (*n* = 42), and those with implausible energy intake (*n* = 1), resulting in the final inclusion of 14,553 participants. For the analysis of Yamada-type polyps, 2,958 participants were excluded due to missing Yamade type data, leaving 11,595 participants. In the analysis of cooking methods of soys, 5,906 participants were included, with 8,647 excluded due to missing cooking methods data (Figure 1).

### 2.2 Dietary assessment and covariates

A validated food frequency questionnaire (FFQ) was used to assess dietary intakes (25). Spearman’s correlation coefficient for soy intake is 0.3, which means that they moderately correlated. The FFQ covered 15 food categories, including rice, wheat, other staple foods (e.g., corn, millet, black rice, etc.), unprocessed red meat, processed red meat, poultry, fish or seafood, eggs, fresh vegetables, soy products, preserved vegetables, fresh fruit, milk or fermented dairy products (e.g., cheese, yogurt, etc.), and spicy foods. Consumption frequency was categorized into five levels (never, daily, weekly, monthly, or yearly). Additionally, we gathered the information on the cooking methods of soy products, including the frequency and intake of boiled soys (white tofu), fried soys (braised, stir-fried, or stewed tofu), and marinated soys (pickled dried tofu). Trained interviewers conducted in-person questionnaires, providing participants with food model atlases and measuring tools (e.g., glasses, plates, bowls, spoons, etc.) to estimate portion sizes accurately. The FFQ data were converted into dietary nutrient intakes (fat, protein, fiber, and cholesterol) and total energy using The China Food Composition Tables (2018) (26). Furthermore, a Healthy Diet Score was calculated based on 10 major dietary components (24), including fish or seafood intake (> median), fruit intake (> median), vegetable intake (> median), dairy product intake (> median), wholegrain intake (> median), tea intake (> median), processed red meat intake (≤ median), unprocessed red meat intake (≤ median), preserved vegetable intake (≤ median), and refined grain intake (≤ median). Each favorable dietary factor contributed one point, with higher scores indicating a healthier diet within the 0–10 score range.

Demographic characteristics (age, sex, education level, and annual household income), personal and family history of CRC, lifestyle factors (physical activity, smoking status, and alcohol consumption), and medication use (aspirin, vitamins, and calcium supplements) were collected by trained medical staffs using detailed questionnaires. Body weight and height were measured anthropometrically, and BMI was calculated as weight (kg) divided by height (m) squared. According to Chinese standards, participants were categorized as underweight (< 18.5 kg/m^2^), normal weight (18.5–23.9 kg/m^2^), overweight (24–27.9 kg/m^2^), and obese (≥ 28 kg/m^2^) (27, 28). Weekly physical activity levels were calculated in metabolic equivalent task hours (MET-h per week) using the Compendium of Physical Activities (29).

### 2.3 Outcome ascertainment

Anatomic subsite categorization was based on the location of polyps: those found in the cecum, ascending colon, hepatic flexure, transverse colon, or splenic flexure were considered proximal, while those in the descending or sigmoid colon were classified as distal, and those in the rectum or rectosigmoid junction were designated as rectal. Additionally, colorectal polyps were graded by size into small (diameter < 10 mm) and large (diameter ≥ 10 mm) categories (5). Multiplicity was classified into solitary (quantity = 1) and multiple (quantity >1). Polyps were further categorized by Yamada type, with those ≤ II indicating a diagnosis of Yamada I or II, and those ≥ III indicating a diagnosis of Yamada III or IV at least once (30).

### 2.4 Statistical analysis

The intake of foods is expressed in terms of energy density (g·2,000 kcal^−1^·d^−1^) using the nutrient density method (31). Baseline characteristics are described with continuous variables presented as means ± standard errors, and categorical variables denoted by numeric values accompanied by percentages (%).

Multivariate regression analysis was used to derive odds ratios (ORs) and 95% confidence intervals (95% CIs) for the association between soy products and the prevalence of colorectal polyps and adenoma. Known risk factors were incorporated as covariates within the models, and a stepwise regression approach was employed. Model 1 was age- and sex-adjusted. Model 2 included additional adjustments for BMI (< 18.5, 18.5–24, 24–28, > 28, in kg/m^2^), smoking (never, past smokers with <25 pack-years or ≥ 25 pack-years, current smokers with <25 pack-years or ≥ 25 pack-years), alcohol consumption (never, ≤ 25 mL/day for men and ≤ 15 mL/day for women, or > 25 mL/day for men and > 15 mL/day for women), household annual income (yuan), physical activity (MET-h per week), vitamin supplement use (yes or no), history of family CRC (yes or no), regular aspirin use (yes or no), and educational level (< middle school or ≥ middle school). Building on model 2, model 3 was further adjusted for total energy intake (quartile), intake of fresh vegetable (quartile), intake of fruit (quartile) and intake of unprocessed red meat (quartile). Model 4 was further adjusted for total energy intake (quartile) and healthy diet score (quartile) based on model 2. Median values of the respective categories were employed as a continuous variable to test the linear trend.

In the secondary analysis, ORs and 95% CIs were calculated for polyps with varying histopathological features, including size, multiplicity, Yamada type, and anatomic location. The association between soy products and colorectal polyps and adenomas was further evaluated, considering cooking methods. Soy products were categorized into three groups based on culinary preparation: boiled soy, fried soy (including braised, stir-fried, and stewed), and marinated soy. Subtype analysis, sensitivity analysis, and subgroup analysis were conducted in populations with cooking information.

Sensitivity analyses were performed to assess model robustness, excluding individuals with extreme energy intake (< 800 kcal/day or > 4,200 kcal/day for males and < 600 kcal/day or > 3,500 kcal/day for females), those with extreme BMI values (< 15 or > 40 kg/m^2^), and further adjusting for calcium supplement intake and baseline diabetes status based on model 4. Subgroup analysis involved stratification based on covariates such as age, BMI, sex, physical activity, smoking status, alcohol intake, education, total energy intake, and healthy diet score to explore potential differential outcomes.

Statistical analysis was conducted using SAS 9.4, considering a correlation as significant when the bilateral *p*-value was <0.05.

## 3 Results

### 3.1 Characteristics

Between March 2018 and December 2022, among 14,553 participants, a total of 4,942 cases of colorectal polyps and 2,678 cases of colorectal adenomas were documented. In this high-risk population, the median intake of soy products was 18.76 g/2,000 kcal per day. Baseline characteristics revealed that individuals with higher consumption of soy products tended to be female and more educated, with a higher household income. Furthermore, this group was more likely to take vitamin and calcium supplements. However, they were less likely to engage in physical activity and consume alcohol. Regarding dietary habits, individuals with higher soy consumption exhibited lower intake of grains and unprocessed red meat, while their consumption of seafood, poultry, and processed red meat seemed higher. These participants also showed higher healthy diet scores (Table 1).

**Table 1:** Baseline characteristics of study participants according to quartiles of soy consumption^a^.

Characteristics	Quartiles of soy consumption (g·2,000 kcal^−1^·d^−1^)			
	Q1^b^	Q2	Q3	Q4
N	3,638	3,638	3,639	3,638
Range (g·2,000 kcal^−1^·d^−1^)	<8.02	8.02 ~ 18.76	18.77 ~ 38.46	≥38.46
Age (years)	60.77 ± 7.86	60.00 ± 7.91	60.46 ± 7.98	60.85 ± 7.82
Sex, n (%)				
Male	1,916 (52.67)	1,904 (52.34)	1,949 (53.56)	1,833 (50.38)
Female	1,722 (47.33)	1,734 (47.66)	1,690 (46.44)	1,805 (49.62)
Family history of colorectal cancer, n (%)				
No	3,297 (90.63)	3,326 (91.42)	3,321 (91.26)	3,312 (91.04)
Yes	225 (6.18)	186 (5.11)	227 (6.24)	250 (6.87)
Body mass index (BMI) (kg/m^2^)				
<18.5, n (%)	132 (3.63)	157 (4.32)	111 (3.05)	110 (3.02)
18.5–24.0, n (%)	1,960 (53.88)	1,959 (53.85)	1,928 (52.98)	1,887 (51.87)
24.0–28, n (%)	1,254 (34.47)	1,255 (34.50)	1,326 (36.44)	1,333 (36.64)
>28, n (%)	292 (8.03)	267 (7.34)	274 (7.53)	308 (8.47)
Educational level				
< middle school, n (%)	3,288 (90.38)	3,134 (86.15)	3,044 (83.65)	2,974 (81.75)
≥ middle school, n (%)	350 (9.62)	504 (13.85)	595 (16.35)	664 (18.25)
Household income (yuan per year), n (%)				
< 30,000	1,418 (38.98)	1,222 (33.59)	1,123 (30.86)	1,052 (28.92)
30,000–100,000	1,480 (40.68)	1,544 (42.44)	1,575 (43.28)	1,612 (44.31)
> 100,000	664 (18.25)	779 (21.41)	884 (24.29)	944 (25.95)
Physical activity (MET-h per week ^c^)				
Q1 (<75.0), n (%)	830 (22.81)	779 (21.41)	1,011 (27.78)	1,023 (28.12)
Q2 (75.3 ~ 139.5), n (%)	884 (24.30)	900 (24.74)	906 (24.90)	943 (25.92)
Q3 (140.0 ~ 253.8), n (%)	829 (22.79)	900 (24.74)	956 (26.27)	959 (26.36)
Q4 (≥254.0), n (%)	1,095 (30.10)	1,059 (29.11)	766 (21.05)	713 (19.60)
Smoke, n (%)				
Never	2,310 (63.50)	2,329 (64.02)	2,320 (63.75)	2,325 (63.91)
Past smokers				
<25 pack-years	298 (8.19)	337 (9.26)	270 (7.42)	295 (8.11)
≥25 pack-years	256 (7.04)	206 (5.66)	240 (6.60)	253 (6.95)
Current smokers				
<25 pack-years	304 (8.36)	301 (8.27)	307 (8.44)	285 (7.83)
≥25 pack-years	470 (12.92)	465 (12.78)	502 (13.79)	480 (13.19)
Alcohol drinker, n (%)				
Never	2,165 (59.51)	2,059 (56.60)	2,113 (58.07)	2,230 (61.30)
≤25 mL/d for men, ≤15 mL/d for women	225 (6.18)	243 (6.68)	250 (6.87)	239 (6.57)
>25 mL/d for men, >15 mL/d for women	1,248 (34.30)	1,336 (36.72)	1,276 (35.06)	1,169 (32.13)
Vitamin supplement intake, n (%)				
No	3,602 (99.01)	3,582 (98.46)	3,574 (98.21)	3,582 (98.46)
Yes	36 (0.99)	56 (1.54)	65 (1.79)	56 (1.54)
Calcium supplement intake, n (%)				
No	3,296 (90.60)	3,268 (89.83)	3,212 (88.27)	3,185 (87.55)
Yes	342 (9.40)	370 (10.17)	427 (11.73)	453 (12.45)
Regular aspirin use, n (%)				
No	3,591 (98.71)	3,586 (98.57)	3,569 (98.08)	3,547 (97.50)
Yes	45 (1.24)	52 (1.43)	69 (1.90)	91 (2.50)
Cancer, n (%)	55 (1.51)	61 (1.68)	56 (1.54)	58 (1.59)
Dietary intake				
Total energy (kcal·d^−1^)	2,134.15 ± 788.21	2,103.66 ± 758.98	2,148.00 ± 754.93	2,164.72 ± 796.70
Whole grains (g·2,000 kcal^−1^·d^−1^)	443.58 ± 147.40	431.05 ± 140.53	436.14 ± 137.87	405.03 ± 133.96
Refined grains (g·2,000 kcal^−1^·d^−1^)	438.27 ± 147.11	425.34 ± 140.48	428.98 ± 137.62	396.84 ± 134.52
Total dairy products (g·2,000 kcal^−1^·d^−1^)	35.48 ± 81.28	35.08 ± 76.86	34.43 ± 75.86	34.13 ± 72.51
Fresh vegetables (g·2,000 kcal^−1^·d^−1^)	218.70 ± 114.26	220.59 ± 103.95	221.56 ± 107.18	232.38 ± 115.41
Preserved vegetables (g·2,000 kcal^−1^·d^−1^)	4.82 ± 12.03	4.66 ± 13.18	4.92 ± 9.86	6.74 ± 14.95
Fruits (g·2,000 kcal^−1^·d^−1^)	90.22 ± 115.83	96.79 ± 94.52	93.51 ± 96.40	94.73 ± 97.48
Tea (g·2,000 kcal^−1^·d^−1^)	446.55 ± 1,131.89	427.42 ± 1,169.98	422.63 ± 1,148.65	477.14 ± 1,293.24
Seafood (g·2,000 kcal^−1^·d^−1^)	14.19 ± 21.52	14.61 ± 18.96	17.86 ± 26.76	18.64 ± 26.14
Poultry (g·2,000 kcal^−1^·d^−1^)	4.74 ± 10.30	5.05 ± 8.43	5.82 ± 9.37	6.50 ± 12.80
Processed red meat (g·2,000 kcal^−1^·d^−1^)	0.61 ± 3.25	0.60 ± 1.91	0.79 ± 3.43	0.86 ± 2.85
Unprocessed red meat (g·2,000 kcal^−1^·d^−1^)	53.01 ± 57.41	50.63 ± 46.54	43.43 ± 41.03	39.22 ± 37.51
Healthy diet score	4.79 ± 1.62	4.93 ± 1.63	4.95 ± 1.65	5.12 ± 1.71

^a^Data are percentages or means (standard errors) unless indicated otherwise. ^b^Q, quartile. ^c^MET-h per week: metabolic equivalent task hours per week.

### 3.2 Soy consumption and colorectal polyp and adenoma prevalence

In the age- and sex-adjusted Model 1, a significant positive association between soy consumption and the prevalence of colorectal polyps was observed [OR (95% CI) Q3 vs. Q1: 1.15 (1.04–1.27); OR (95% CI) Q4 vs. Q1: 1.16 (1.05–1.29); P-trend <0.001]. This association persisted after adjustment for demographic characteristics in Model 2 [OR (95% CI) Q3 vs. Q1: 1.12 (1.01–1.24); OR (95% CI) Q4 vs. Q1: 1.12 (1.01–1.24); P-trend = 0.007]. The positive correlation remained consistent in Models 3 [OR (95% CI) Q3 vs. Q1: 1.12 (1.01–1.24); OR (95% CI) Q4 vs. Q1: 1.11 (1.00–1.23); P-trend = 0.014] and 4 [OR (95% CI) Q3 vs. Q1: 1.12 (1.01–1.24); OR (95% CI) Q4 vs. Q1: 1.12 (1.01–1.24); P-trend = 0.007]. Models 3 and 4 were built on Model 2, incorporating adjustment for energy and dietary intake, and energy and healthy diet score, respectively (Table 2).

**Table 2:** Multivariable-adjusted ORs (95% CIs) of the prevalence of colorectal polyps or adenomas according to quartiles of soy consumption^a^.

	Quartiles of soy consumption (g·2,000 kcal^−1^·d^−1^)				*p*-trend
	Q1	Q2	Q3	Q4	
Range (g·2,000 kcal^−1^·d^−1^)	<8.02	8.02 ~ 18.76	18.77 ~ 38.46	≥38.46	
Polyps					
Cases/n	1,190/3,638	1,162/3,638	1,294/3,639	1,296/3,638	
Model 1^b^	1 (Ref.)	1.00 (0.90–1.10)	1.15 (1.04–1.27)	1.16 (1.05–1.29)	<0.001
Model 2^c^	1 (Ref.)	1.00 (0.90–1.11)	1.12 (1.01–1.24)	1.12 (1.01–1.24)	0.007
Model 3^d^	1 (Ref.)	1.01 (0.91–1.11)	1.12 (1.01–1.24)	1.11 (1.00–1.23)	0.014
Model 4^e^	1 (Ref.)	1.00 (0.91–1.11)	1.12 (1.01–1.24)	1.12 (1.01–1.24)	0.007
Adenomas					
Cases/n	637/3,638	604/3,638	709/3,639	728/3,638	
Model 1	1 (Ref.)	0.96 (0.85–1.09)	1.15 (1.02–1.29)	1.20 (1.06–1.35)	<0.001
Model 2	1 (Ref.)	0.97 (0.85–1.10)	1.13 (1.00–1.27)	1.17 (1.03–1.32)	0.002
Model 3	1 (Ref.)	0.97 (0.86–1.10)	1.11 (0.98–1.26)	1.14 (1.01–1.29)	0.008
Model 4	1 (Ref.)	0.97 (0.86–1.10)	1.12 (0.99–1.27)	1.16 (1.03–1.32)	0.003

^a^Q, quartile. ^b^Model 1 was adjusted for age and sex. ^c^Model 2 was adjusted for model 1 plus BMI (<18.5, 18.5–24, 24–28, >28, in kg/m^2^), smoking (never, past smokers with <25 pack-years or ≥ 25 pack-years, current smokers with <25 pack-years or ≥ 25 pack-years), alcohol consumption (never, ≤25 mL/day for men and ≤ 15 mL/day for women, >25 mL/day for men and > 15 mL/day for women), household annual income (yuan), physical activity (MET-h per week), vitamin supplement use (yes or no), history of family colorectal cancer (yes or no), regular aspirin use (yes or no), educational level (< middle school or ≥ middle school). ^d^Model 3 was adjusted for model 2 plus total energy intake (quartile), intake of fresh vegetable (quartile), intake of fruit (quartile), intake of unprocessed red meat (quartile). ^e^Model 4 was adjusted for model 2 plus total energy intake (quartile), healthy diet score (quartile).

Further analysis of colorectal polyp subtypes, considering size, multiplicity, Yamada classification, and anatomical location, revealed that higher soy intake was associated with the occurrence of polyps ≥10 mm, multifocal polyps, Yamada type II or less, and exhibited positive correlations across all anatomical locations (distal colon, proximal colon, and rectum; Supplementary Table 2).

The relationship between soy intake and the prevalence of colorectal adenomas was also significant. Using the lowest quartile of soy intake as the reference, the ORs (95% CIs) for the highest quartile were 1.20 (1.06–1.35) in Model 1 (*p*-trend <0.001), 1.17 (1.03–1.32) in Model 2 (*p*-trend = 0.002), 1.14 (1.01–1.29) in Model 3 (*p*-trend = 0.008), and 1.16 (1.03–1.32) in Model 4 (*p*-trend = 0.003; Table 2).

### 3.3 Associations of soys by different cooking methods with colorectal polyp and adenoma prevalence

**Figure 2:**
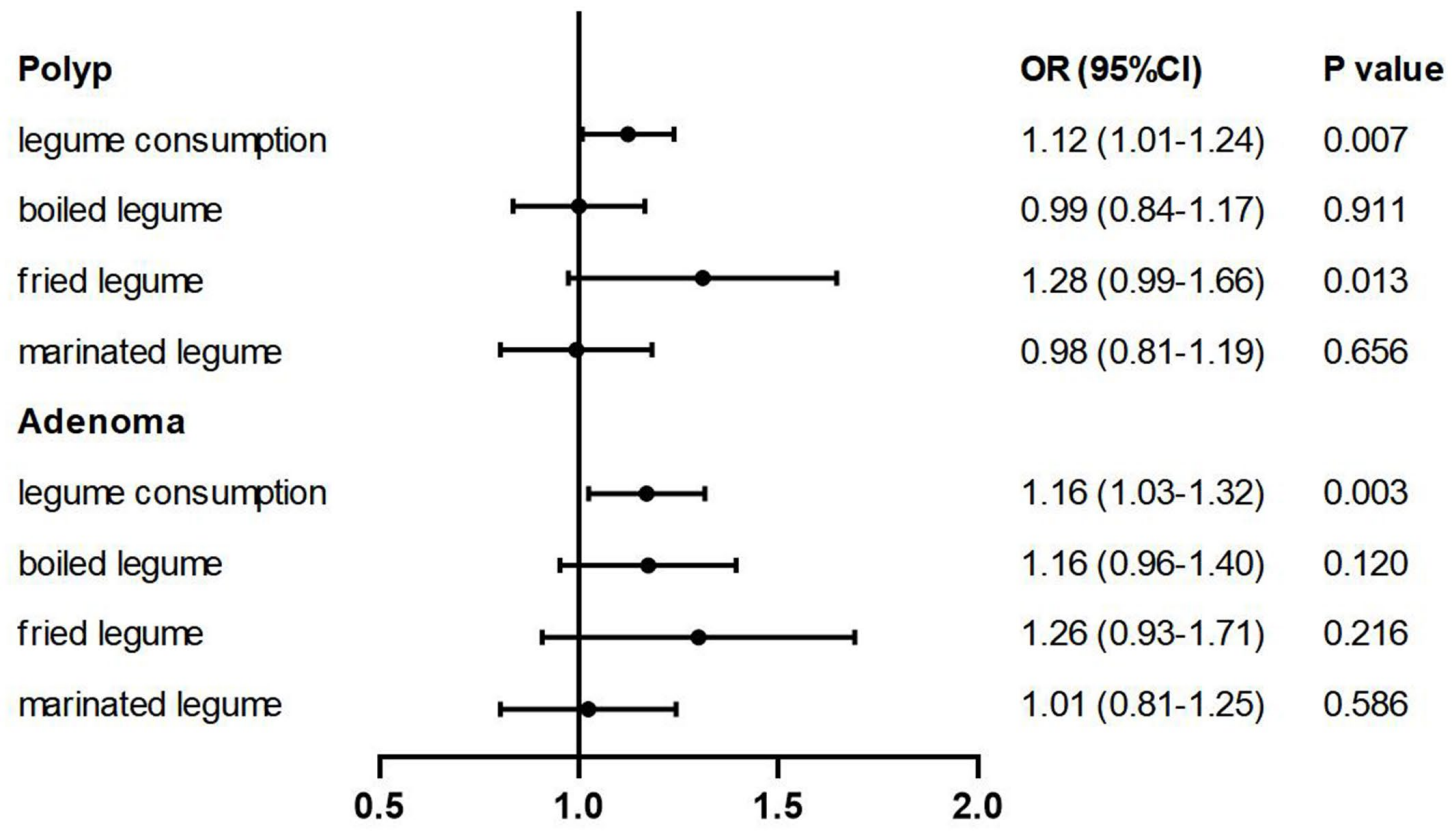
Multivariable-adjusted ORs (95% CIs) of the prevalence of colorectal polyps/adenomas according to soy intake and soy products by different cooking methods. The model was adjusted for age, sex, BMI (<18.5, 18.5–24, 24–28, >28, in kg/m^2^), smoking (never, past smokers with <25 pack-years or ≥ 25 pack-years, current smokers with <25 pack-years or ≥ 25 pack-years), alcohol consumption (never, ≤25 mL/day for men and ≤ 15 mL/day for women, >25 mL/day for men and > 15 mL/day for women), household annual income (yuan), physical activity (MET-h per week), vitamin supplement use (yes or no), history of family colorectal cancer (yes or no), regular aspirin use (yes or no), educational level (< middle school or ≥ middle school), total energy intake (quartile) and healthy diet score (quartile).

In 5,906 participants consuming soys with different cooking methods, 4,294 people never consumed boiled soys and 4,792 did not like marinated soys, while only 316 individuals with no fried soy consumption (Supplementary Table 1). After categorizing soy products into three groups based on cooking methods—boiled, fried, and marinated soys—our findings revealed that the consumption of fried soy products exceeding 25.60 g per 2,000 kcal per day is associated with a 1.3-fold increase in the prevalence of colorectal polyps compared to non-consumers. As shown in Table 3, the number of people consuming fried soys was 3.5 times that of those consuming boiled beans and 5 times that of those consuming marinated beans. Among those with non-zero soy intake, the median intake of fried soys was about 6–8 times higher than that of the other two categories. The ORs (95% CIs) were 1.36 (1.06–1.76) in Model 1 (*p*-trend = 0.003), 1.29 (0.99–1.67) in Model 2 (*p*-trend = 0.010), 1.27 (0.98–1.65) in Model 3 (*p*-trend = 0.013), and 1.28 (0.99–1.66) in Model 4 (*p*-trend = 0.013). No significant associations were observed for boiled or marinated soys (Figure 2; Table 3).

**Table 3:** Multivariable-adjusted ORs (95% CIs) of the prevalence of colorectal polyps or adenomas according to the intake of soy products by different cooking methods^a^.

	Classification of soy consumption (g·2,000 kcal^−1^·d^−1^)			*p*-trend
	C1	C2	C3	
Boiled soys				
Range (g·2,000 kcal^−1^·d^−1^)	0.00	0.00 ~ 4.40	>4.40	
Polyps				
Cases/n	1,546/4,294	317/806	304/806	
Model 1^b^	1 (Ref.)	1.10 (0.94–129)	1.06 (0.91–1.25)	0.286
Model 2^c^	1 (Ref.)	1.00 (0.85–1.19)	1.00 (0.84–1.18)	0.967
Model 3^d^	1 (Ref.)	1.00 (0.84–1.18)	0.99 (0.84–1.18)	0.933
Model 4^e^	1 (Ref.)	1.00 (0.84–1.18)	0.99 (0.84–1.17)	0.911
Adenomas				
Cases/n	903/4,294	193/806	198/198	
Model 1	1 (Ref.)	1.15 (0.96–1.38)	1.23 (1.02–1.47)	0.014
Model 2	1 (Ref.)	1.08 (0.89–1.30)	1.17 (0.97–1.41)	0.100
Model 3	1 (Ref.)	1.07 (0.89–1.30)	1.16 (0.96–1.40)	0.116
Model 4	1 (Ref.)	1.07 (0.88–1.29)	1.16 (0.96–1.40)	0.120
Fried soys				
Range (g·2,000 kcal^−1^·d^−1^)	0.00	0.00 ~ 25.60	>25.60	
Polyps				
Cases/n	105/316	989/2,795	1,073/2,795	
Model 1	1 (Ref.)	1.19 (0.93–1.54)	1.36 (1.06–1.76)	0.003
Model 2	1 (Ref.)	1.14 (0.88–1.47)	1.29 (0.99–1.67)	0.010
Model 3	1 (Ref.)	1.12 (0.86–1.45)	1.27 (0.98–1.65)	0.013
Model 4	1 (Ref.)	1.13 (0.87–1.46)	1.28 (0.99–1.66)	0.013
Adenomas				
Cases/n	60/316	605/2,795	629/2,795	
Model 1	1 (Ref.)	1.28 (0.95–1.72)	1.34 (0.99–1.81)	0.103
Model 2	1 (Ref.)	1.21 (0.90–1.64)	1.26 (0.93–1.71)	0.220
Model 3	1 (Ref.)	1.19 (0.88–1.62)	1.24 (0.91–1.68)	0.247
Model 4	1 (Ref.)	1.21 (0.90–1.64)	1.26 (0.93–1.71)	0.216
Marinated soys				
Range (g·2,000 kcal^−1^·d^−1^)	0.00	0.00 ~ 3.19	≥3.19	
Polyps				
Cases/n	1,722/4,792	236/557	209/557	
Model 1	1 (Ref.)	1.28 (1.06–1.53)	1.04 (0.86–1.25)	0.196
Model 2	1 (Ref.)	1.25 (1.03–1.51)	0.98 (0.81–1.19)	0.522
Model 3	1 (Ref.)	1.23 (1.01–1.48)	0.98 (0.81–1.19)	0.564
Model 4	1 (Ref.)	1.23 (1.02–1.49)	0.98 (0.81–1.19)	0.656
Adenomas				
Cases/n	1,026/4,792	140/557	128/557	
Model 1	1 (Ref.)	1.21 (0.98–1.49)	1.08 (0.87–1.33)	0.212
Model 2	1 (Ref.)	1.15 (0.93–1.43)	1.02 (0.82–1.27)	0.549
Model 3	1 (Ref.)	1.12 (0.91–1.40)	1.01 (0.82–1.26)	0.624
Model 4	1 (Ref.)	1.13 (0.91–1.40)	1.01 (0.81–1.25)	0.586

^a^C, classification. ^b^Model 1 was adjusted for age and sex. ^c^Model 2 was adjusted for model 1 plus BMI (< 18.5, 18.5–24, 24–28, > 28, in kg/m2), smoking (never, past smokers with <25 pack-years or ≥ 25 pack-years, current smokers with <25 pack-years or ≥ 25 pack-years), alcohol consumption (never, ≤ 25 mL/day for men and ≤ 15 mL/day for women, > 25 mL/day for men and > 15 mL/day for women), household annual income (yuan), physical activity (MET-h per week), vitamin supplement use (yes or no), history of family colorectal cancer (yes or no), regular aspirin use (yes or no), educational level (<middle school or ≥ middle school) and bean consumption of remaining cooking methods (boiled soy, fried soy, marinated soy, remaining soy). ^d^Model 3 was adjusted for model 2 plus total energy intake (quartile), intake of fresh vegetable (quartile), intake of fruit (quartile), intake of unprocessed red meat (quartile). ^e^Model 4 was adjusted for model 2 plus total energy intake (quartile), healthy diet score (quartile).

In subtype analyses, the impact of cooking methods was further considered. After multivariable adjustment, the prevalence of rectum polyps was higher among the highest consumers of boiled soy products [OR (95% CI) Q2 vs. Q1: 1.34 (1.01–1.79); OR (95% CI) Q3 vs. Q1: 1.37 (1.02–1.82); *p*-trend = 0.014]. Moreover, the risk of developing polyps ≥10 mm in diameter (*p*-trend = 0.030), multifocal polyps (*p*-trend = 0.017), and polyps of Yamada type II or less (*p*-trend = 0.011) increased with greater consumption of fried soys. There was also a heightened risk for polyps located in the proximal (*p*-trend = 0.024) and distal colon (*p*-trend = 0.030; Supplementary Table 3). We further supplemented our analysis with an examination of the intake of other foods prepared using different cooking methods, such as poultry, eggs, and fish. The findings revealed a positive correlation solely between the consumption of fried soys and marinated eggs with polyps/adenomas (Supplementary Table 4).

### 3.4 Subgroup analyses and sensitivity analyses

In subgroup analyses, no significant interactions were observed when stratified by baseline age, BMI, sex, MET, smoking, alcohol, education, energy intake, and healthy diet score, whether considering cooking methods or not (Supplementary Tables 5, 6). To assess the reliability of the results, sensitivity analyses were conducted by excluding individuals with extreme energy intake and extreme BMIs, and further adjustment for calcium supplement use and baseline diabetes. Whether or not considering cooking methods, the positive association between the intake of soy products/fried soys and the prevalence of colorectal polyps or adenomas did not undergo any substantial changes, confirming the robustness of our model (Supplementary Tables 7, 8).

## 4 Discussion

In this study, an extension of previous data to include participants meeting the criteria from 2019 to 2022 revealed a significant positive association between the consumption of soy products and the prevalence of colorectal polyps and adenomas among high-risk participants in the LP3C study. This correlation was particularly notable for large, multiple, and Yamada type II or lower polyps. Further classification based on cooking methods indicated that the potential hazardous effects were predominantly associated with fried soys.

However, the conclusions drawn have not been consistent. For instance, among Chinese males without CRC, a follow-up of 390,688 person-years revealed no significant correlation between legume intake and the risk of CRC (32). In postmenopausal women, the consumption of soy foods may reduce the risk of CRC (33), while a similar inverse association has been observed between the intake of soy foods and the risk of proximal colon cancer in men (34). These studies were conducted in Shanghai or Japan, both Asian populations, but the dietary patterns in these areas differ from those in Lanxi, with a wider variety of soy products consumed, including soy milk, fresh green soy beans, and bean sprouts (33). Residents in Lanxi tend to prefer a diet high in oil and salt with strong flavors, thus highlighting the role of fried soy products in our population. Moreover, meta-analyses of prospective studies have also yielded conflicting results. One that included 14 cohort studies indicated that higher legume consumption was associated with a reduced risk of CRC (16), while the other pooled analysis showed no significant evidence in Asian populations (17). Meta-analyses or pooled analyses, despite larger sample sizes, often involve more diverse populations and a greater variety of legume products, which might obscure the harmful effects of fried soy products.

We hypothesize that the antinutrients in soys may affect the bioavailability of trace elements (35), thereby influencing colorectal health. However, we contend that the positive correlation observed between higher soy intake and the prevalence of colorectal polyps/adenomas in this study is more likely attributable to the cooking method of high-temperature frying.

In our investigation, the predominant soy consumed was soy-based products, such as tofu. Frying entails the incorporation of quantities of oil, whereas ingredients added during boiling predominantly consist of water, and the processes of marinating tend to result in increased sodium intake. Our findings uncover a positive association between the intake of soy products and the prevalence of colorectal polyps or adenomas, identifying a potential hazard originating from fried soys, which constitute the majority of soys consumed in our study. Individuals consuming fried soy products were approximately 3.5 times more numerous than those preferring boiled or marinated soy products, with the median intake of the intake of fried soys being 6–8 times higher (Table 2). The positive correlation between the intake of fried soys and the prevalence of polyps may largely account for the relationship of total intake. Additionally, a similar increased prevalence of adenomas was also observed, although not statistically significant, which was possibly due to the smaller sample size after stratifying. Similar to our study, a previous study suggested fried legume cheese might increase colorectal risk (36). Various cooking conditions, such as pressure and duration, can differently affect the nutrients and antinutrients in legumes (37, 38). The current study primarily identified the hazardous effects of fried legumes on colorectal polyps, indicating that high-temperature frying may alter nutritional content and cause adverse consequences. Fried foods have been identified as one of the key risk factors for colorectal health (8, 21). Therefore, the processing plays a crucial role.

Several potential mechanisms underlie these findings. First, frying foods at high temperatures can produce pollutants such as polycyclic aromatic hydrocarbons and heterocyclic amines, which may increase colorectal risk through DNA damage (39–41). Simultaneously, some nutrients in soys may be reduced or degraded under high-temperature conditions. Second, the frying process often introduces dietary fat, which may alter fecal bile acid (BA) levels, regulate gut microbiota and metabolites, thereby promoting carcinogenesis (21, 42). Under high-fat intake, the generation of BAs and secondary BAs may be stimulated. The former have been found at higher concentrations in the feces of individuals with a higher colorectal risk (43), and prospective studies have shown that plasma BAs are positively associated with the risk of colon cancer (44). Animal models showed that this may act by inducing intestinal microbial dysbiosis, metabolic dysregulation, and increased levels of hemolytic phospholipids (45). Due to residents’ preference for heavily seasoned foods, enhancing its flavor, fried soys often contain more salt, which has been linked to digestive tract cancers, such as gastric cancer (46).

The size and morphology of colorectal polyps are closely associated with their subsequent outcomes (5, 30). In this study, soy consumption was significantly correlated with the prevalence of larger, multiple, and Yamade-typed polyps below stage II, exhibiting positive correlations within the anatomical locations of the proximal colon, distal colon, and rectum, suggesting their severity and universality. Additionally, after stratifying by cooking methods, the association trend between fried soys and its subtypes was roughly consistent with that observed in total soys. The positive correlation observed between boiled soy consumption and rectal polyp prevalence may be explained by the fact that, although the colon and rectum share similar anatomical characteristics and are closely related, they possess distinct functional mechanisms, thus harboring different potential pathophysiological mechanisms (47). Since stool resides longer in the rectum during transport and storage, the concentration of toxic substances may be higher, making the rectum more susceptible to genotoxic and cytotoxic damage (47). Therefore, it is inaccurate to assert that soy itself is detrimental to the colorectum since inappropriate cooking methods may exert a greater negative impact than the foods themselves. Alterations in the inherent nutritional composition of the food, the formation of new contaminants, and the introduction of substances such as oil and salt can all exert diverse effects on the outcomes. Classifying based on cooking methods can provide a more precise analysis of how the process affects the nutrients and how they may influence health.

This study shows several unique strengths. First, it is a pioneering investigation into the relationship between soy products, their culinary methods, and colorectal polyps. Considering cooking methods is an innovative step based on population characteristics and practical applications. Second, compared with previous articles based on the LP3C cohort, the sample size has been expanded, including nearly 15,000 participants with detailed pathological reports of polyps, providing a basis for more detailed subgroup analyses. Furthermore, the FFQ for participants recruited between 2020 and 2022 has been updated to include new questions about cooking methods. Third, we collected crucial exposure and confounding factors such as diet and family history of CRC prior to electronic colonoscopy, thereby minimizing the impact of reverse causality. Additionally, building upon previous research, we separately analyzed polyps and adenomas, offering potential predictions for the progression of CRC.

However, this study has some limitations. First, the inevitable measurement error of self-reported intake affects the true association between soy intake and the prevalence of colorectal polyps. The study was based on unique dietary patterns and lifestyle habits of the Chinese population, making it difficult to generalize the findings widely. The intake type was relatively simple and mainly focused on soy products, which may lead to partial bias. Furthermore, colon polyps exhibited familial aggregation; regrettably, due to the lack of data, genetic factors were not investigated. Nonetheless, family history of CRC was adjusted in our model. Given the cross-sectional nature of our study, the limited ability to explore causality exists. Uncontrollable and unmeasurable confounders can obscure the true relationship. Consequently, longer-term follow-up is required, which is an ongoing endeavor in our subsequent research.

## 5 Conclusion

In conclusion, our research reveals an association between increased consumption of soy products and an elevated prevalence of CRC among high-risk individuals in China, with fried soys emerging as a significant potential hazard. Consequently, the impact of cooking methods on food and disease deserves categorical assessment. The findings suggest that high-risk populations for CRC may minimize the use of high-temperature frying for soys to prevent colorectal polyps and adenomas.

## Data availability statement

The original contributions presented in the study are included in the article/Supplementary material, further inquiries can be directed to the corresponding authors.

## Ethics statement

The studies involving humans were approved by the ethical committee of Lanxi Red Cross Hospital (No. 20180302). The studies were conducted in accordance with the local legislation and institutional requirements. The participants provided their written informed consent to participate in this study.

## Author contributions

WZ: Conceptualization, Data curation, Funding acquisition, Project administration, Resources, Supervision, Writing – review & editing. XuL: Formal analysis, Investigation, Visualization, Writing – original draft, Writing – review & editing. MZ: Formal analysis, Investigation, Writing – review & editing. HY: Formal analysis, Investigation, Visualization, Writing – review & editing. XH: Investigation, Writing – review & editing. XiL: Formal analysis, Writing – review & editing. LH: Investigation, Writing – review & editing. YoZ: Formal analysis, Visualization, Writing – review & editing. PH: Investigation, Writing – review & editing. PZ: Methodology, Software, Validation, Writing – review & editing. JJ: Methodology, Validation, Writing – review & editing. YuZ: Conceptualization, Project administration, Supervision, Writing – review & editing.
