# Supplementary material for: Association of soy intake and cooking methods with colorectal polyp and adenoma prevalence: findings from the extended Lanxi pre-colorectal cancer cohort (LP3C)
Source: Front Nutr. 2024 Jun 19;11:1390143. doi: 10.3389/fnut.2024.1390143 (PMC11221495; doi:10.3389/fnut.2024.1390143)
Supplement: Supplementary file 1 [file Table_1.DOCX]

Supplementary Material

The Supplemental Information includes 8 supplementary tables.

Table of Contents

**Supplementary Table 1.** Way to soy consumption in our population....................................2

**Supplementary Table 2.** Multivariable-adjusted ORs (95% CIs) of soy consumption with the prevalence of polyps according to subtypes...............................................................……..3

**Supplementary Table 3.** Multivariable-adjusted ORs (95% CIs) of soy consumption in different cooking methods with the prevalence of polyps according to subtypes….................4

**Supplementary Table 4.** Multivariable-adjusted ORs (95% CIs) of other food by different cooking methods with the prevalence of colorectal polyps or adenomas………......................6

**Supplementary Table 5.** Subgroup analyses for multivariable-adjusted ORs (95% CIs) of soy consumption with the prevalence of polyps or adenomas...................................................7

**Supplementary Table 6.** Subgroup analyses for multivariable-adjusted ORs (95% CIs) of fried soy consumption with the prevalence of polyps or adenomas…................................…10

**Supplementary Table 7.** Sensitivity analyses for the multivariable-adjusted ORs (95% CIs) of colorectal polyp or adenoma prevalence according to soy consumption............................13

**Supplementary Table 8.** Sensitivity analyses for the multivariable-adjusted ORs (95% CIs) of colorectal polyp or adenoma prevalence according to soy consumption in different cooking methods.…………......................................................................................…..............….......14

**Supplementary table 1**. Way to soy consumption in our population.

|  | All | Non-consumer | Consumer |
| --- | --- | --- | --- |
| **Boiled soy** |  |  |  |
| Mean intake (g·2,000 kcal^-1^·d^-1^) | 3.07 | 0 | 11.24 |
| N(%) | 5906 | 4,294 (72.71%) | 1,612 (27.29%) |
| **Fried soy** |  |  |  |
| Mean intake (g·2,000 kcal^-1^·d^-1^) | 32.98 | 0 | 0.192 |
| N(%) | 5906 | 316 (5.35%) | <0.001 |
| **Marinated soy** |  |  |  |
| Mean intake (g·2,000 kcal^-1^·d^-1^) | 1.46 | 0 | 7.76 |
| N(%) | 5906 | 4,792 (81.14%) | 1,114 (18.86%) |

**Supplementary table 2**. Multivariable-adjusted ORs (95% CIs) of soy consumption with the prevalence of polyps according to subtypes ^a^.

| Subtypes |  | Quartiles of soy consumption (g·2,000 kcal^-1^·d^-1^) | | | | P-trend |
| --- | --- | --- | --- | --- | --- | --- |
|  |  | Q1 | Q2 | Q3 | Q4 |  |
| Size |  |  |  |  |  |  |
| <10 mm | Cases | 945 | 931 | 1,002 | 1,003 |  |
|  | OR (95% CI) | 1 (Ref.) | 1.01 (0.90-1.12) | 1.09 (0.98-1.22) | 1.09 (0.98-1.22) | 0.058 |
| ≥10 mm | Cases | 244 | 230 | 292 | 292 |  |
|  | OR (95% CI) | 1 (Ref.) | 1.00 (0.82-1.21) | 1.24 (1.03-1.49) | 1.25 (1.04-1.50) | 0.004 |
| Multiplicity |  |  |  |  |  |  |
| Single | Cases | 805 | 805 | 873 | 814 |  |
|  | OR (95% CI) | 1 (Ref.) | 1.02 (0.91-1.15) | 1.13 (1.00-1.26) | 1.05 (0.94-1.18) | 0.192 |
| Multiple | Cases | 385 | 357 | 421 | 482 |  |
|  | OR (95% CI) | 1 (Ref.) | 0.96 (0.82-1.13) | 1.10 (0.94-1.29) | 1.28 (1.09-1.49) | <0.001 |
| Yamada type |  |  |  |  |  |  |
| ≤ II | Cases | 583 | 508 | 709 | 779 |  |
|  | OR (95% CI) | 1 (Ref.) | 0.96 (0.84-1.11) | 1.09 (0.95-1.24) | 1.11 (0.97-1.26) | 0.046 |
| ≥ III | Cases | 370 | 309 | 425 | 409 |  |
|  | OR (95% CI) | 1 (Ref.) | 0.94 (0.79-1.11) | 1.04 (0.89-1.22) | 0.95 (0.81-1.12) | 0.813 |
| Anatomic Subsite |  |  |  |  |  |  |
| Distal colon | Cases | 603 | 560 | 692 | 682 |  |
|  | OR (95% CI) | 1 (Ref.) | 0.95 (0.83-1.08) | 1.17 (1.03-1.33) | 1.14 (1.01-1.30) | 0.012 |
| Proximal colon | Cases | 524 | 513 | 556 | 600 |  |
|  | OR (95% CI) | 1 (Ref.) | 1.02 (0.89-1.17) | 1.09 (0.95-1.25) | 1.18 (1.03-1.35) | 0.004 |
| Rectum | Cases | 197 | 221 | 259 | 253 |  |
|  | OR (95% CI) | 1 (Ref.) | 1.16 (0.94-1.42) | 1.35 (1.11-1.64) | 1.32 (1.08-1.62) | 0.002 |

^a^ Q, quartile; Multivariable model adjusted for age, sex, BMI, smoking, alcohol consumption, household annual income, physical activity, vitamin supplement use, history of family colorectal cancer, regular aspirin use, educational level, total energy intake and healthy diet score.

**Supplementary table 3.** Multivariable-adjusted ORs (95% CIs) of soy consumption in different cooking methods with the prevalence of polyps according to subtypes ^a^.

| Subtypes |  | Quartiles of soy consumption  (g·2,000 kcal^-1^·d^-1^) | | | P-trend |
| --- | --- | --- | --- | --- | --- |
|  |  | C1 | C2 | C3 |  |
| Boiled soys |  |  |  |  |  |
| Size |  |  |  |  |  |
| <10 mm | Cases | 1,115 | 227 | 221 |  |
|  | OR (95% CI) | 1 (Ref.) | 1.00 (0.83-1.21) | 1.02 (0.84-1.22) | 0.874 |
| ≥10 mm | Cases | 431 | 90 | 83 |  |
|  | OR (95% CI) | 1 (Ref.) | 0.98 (0.75-1.28) | 0.93 (0.71-1.22) | 0.603 |
| Multiplicity |  |  |  |  |  |
| Single | Cases | 945 | 187 | 170 |  |
|  | OR (95% CI) | 1 (Ref.) | 1.02 (0.84-1.25) | 0.95 (0.77-1.16) | 0.681 |
| Multiple | Cases | 601 | 130 | 134 |  |
|  | OR (95% CI) | 1 (Ref.) | 0.96 (0.76-1.21) | 1.06 (0.84-1.33) | 0.749 |
| Yamada type |  |  |  |  |  |
| ≤ II | Cases | 973 | 210 | 189 |  |
|  | OR (95% CI) | 1 (Ref.) | 1.04 (0.86-1.26) | 0.97 (0.80-1.18) | 0.908 |
| ≥ III | Cases | 573 | 107 | 115 |  |
|  | OR (95% CI) | 1 (Ref.) | 0.91 (0.71-1.16) | 1.03 (0.81-1.30) | 0.977 |
| Anatomic Subsite |  |  |  |  |  |
| Distal colon | Cases | 899 | 186 | 168 |  |
|  | OR (95% CI) | 1 (Ref.) | 0.93 (0.76-1.14) | 0.89 (0.72-1.09) | 0.216 |
| Proximal colon | Cases | 735 | 139 | 141 |  |
|  | OR (95% CI) | 1 (Ref.) | 0.94 (0.75-1.17) | 0.98 (0.79-1.22) | 0.739 |
| Rectum | Cases | 298 | 77 | 76 |  |
|  | OR (95% CI) | 1 (Ref.) | 1.34 (1.01-1.79) | 1.37 (1.02-1.82) | 0.014 |
| Fried soys |  |  |  |  |  |
| Size |  |  |  |  |  |
| <10 mm | Cases | 77 | 714 | 772 |  |
|  | OR (95% CI) | 1 (Ref.) | 1.11 (0.83-1.47) | 1.23 (0.93-1.64) | 0.054 |
| ≥10 mm | Cases | 28 | 275 | 301 |  |
|  | OR (95% CI) | 1 (Ref.) | 1.20 (0.78-1.83) | 1.42 (0.92-2.18) | 0.030 |
| Multiplicity |  |  |  |  |  |
| Single | Cases | 65 | 604 | 633 |  |
|  | OR (95% CI) | 1 (Ref.) | 1.11 (0.82-1.50) | 1.22 (0.90-1.65) | 0.099 |
| Multiple | Cases | 40 | 385 | 440 |  |
|  | OR (95% CI) | 1 (Ref.) | 1.16 (0.80-1.68) | 1.38 (0.95-2.00) | 0.017 |
| Yamada type |  |  |  |  |  |
| ≤ II | Cases | 64 | 617 | 691 |  |
|  | OR (95% CI) | 1 (Ref.) | 1.13 (0.83-1.53) | 1.30 (0.96-1.76) | 0.018 |
| ≥ III | Cases | 41 | 372 | 382 |  |
|  | OR (95% CI) | 1 (Ref.) | 1.14 (0.79-1.65) | 1.25 (0.87-1.81) | 0.149 |
| Anatomic Subsite |  |  |  |  |  |
| Distal colon | Cases | 61 | 570 | 622 |  |
|  | OR (95% CI) | 1 (Ref.) | 1.10 (0.80-1.50) | 1.26 (0.92-1.73) | 0.030 |
| Proximal colon | Cases | 46 | 465 | 504 |  |
|  | OR (95% CI) | 1 (Ref.) | 1.25 (0.89-1.77) | 1.42 (1.00-2.01) | 0.024 |
| Rectum | Cases | 22 | 207 | 222 |  |
|  | OR (95% CI) | 1 (Ref.) | 1.11 (0.69-1.78) | 1.18 (0.73-1.90) | 0.423 |
| Marinated soys |  |  |  |  |  |
| Size |  |  |  |  |  |
| <10 mm | Cases | 1,248 | 168 | 147 |  |
|  | OR (95% CI) | 1 (Ref.) | 1.22 (0.99-1.50) | 0.95 (0.77-1.18) | 0.799 |
| ≥10 mm | Cases | 474 | 68 | 62 |  |
|  | OR (95% CI) | 1 (Ref.) | 1.27 (0.94-1.70) | 1.04 (0.77-1.41) | 0.432 |
| Multiplicity |  |  |  |  |  |
| Single | Cases | 1,043 | 134 | 125 |  |
|  | OR (95% CI) | 1 (Ref.) | 1.20 (0.96-1.50) | 1.02 (0.81-1.27) | 0.478 |
| Multiple | Cases | 679 | 102 | 84 |  |
|  | OR (95% CI) | 1 (Ref.) | 1.27 (0.98-1.64) | 0.91 (0.70-1.20) | 0.965 |
| Yamada type |  |  |  |  |  |
| ≤ II | Cases | 1,090 | 148 | 134 |  |
|  | OR (95% CI) | 1 (Ref.) | 1.22 (0.98-1.51) | 0.99 (0.79-1.23) | 0.607 |
| ≥ III | Cases | 632 | 88 | 75 |  |
|  | OR (95% CI) | 1 (Ref.) | 1.26 (0.97-1.65) | 0.96 (0.73-1.27) | 0.728 |
| Anatomic Subsite |  |  |  |  |  |
| Distal colon | Cases | 967 | 153 | 133 |  |
|  | OR (95% CI) | 1 (Ref.) | 1.42 (1.14-1.77) | 1.10 (0.87-1.37) | 0.073 |
| Proximal colon | Cases | 822 | 102 | 91 |  |
|  | OR (95% CI) | 1 (Ref.) | 1.11 (0.87-1.43) | 0.89 (0.69-1.15) | 0.630 |
| Rectum | Cases | 366 | 44 | 41 |  |
|  | OR (95% CI) | 1 (Ref.) | 1.01 (0.71-1.43) | 0.85 (0.59-1.21) | 0.421 |

^a^ C, classification; Multivariable model adjusted for age, sex, BMI, smoking, alcohol consumption, household annual income, physical activity, vitamin supplement use, history of family colorectal cancer, regular aspirin use, educational level, total energy intake and healthy diet score.

**Supplementary Table 4.** Multivariable-adjusted ORs (95% CIs) of other food by different cooking methods with the prevalence of colorectal polyps or adenomas ^a^.

|  | Classification of food consumption (g·2,000 kcal^-1^·d^-1^) | | | P-trend |
| --- | --- | --- | --- | --- |
|  | C1 | C2 | C3 |  |
| Polyp |  |  |  |  |
| Poultry |  |  |  |  |
| Boiled | 1 (Ref.) | 1.01 (0.87-1.17) | 1.01 (0.88-1.17) | 0.885 |
| Fried | 1 (Ref.) | 1.06 (0.91-1.23) | 1.05 (0.90-1.22) | 0.545 |
| Marinated | 1 (Ref.) | 1.13 (0.95-1.33) | 1.01 (0.85-1.20) | 0.587 |
| Fish |  |  |  |  |
| Boiled | 1 (Ref.) | 1.25 (0.99-1.58) | 1.10 (0.86-1.40) | 0.171 |
| Fried | 1 (Ref.) | 1.03 (0.87-1.23) | 1.01 (0.84-1.20) | 0.937 |
| Egg |  |  |  |  |
| Boiled | 1 (Ref.) | 0.81 (0.68-0.96) | 0.84 (0.70-1.00) | 0.181 |
| Fried | 1 (Ref.) | 1.05 (0.92-1.21) | 0.93 (0.81-1.07) | 0.369 |
| Marinated | 1 (Ref.) | 1.11 (0.96-1.29) | 1.15 (1.00-1.34) | 0.038 |
| soy |  |  |  |  |
| Boiled | 1 (Ref.) | 1.00 (0.84-1.18) | 0.99 (0.84-1.17) | 0.911 |
| Fried | 1 (Ref.) | 1.13 (0.87-1.46) | 1.28 (0.99-1.66) | 0.013 |
| Marinated | 1 (Ref.) | 1.23 (1.02-1.49) | 0.98 (0.81-1.19) | 0.656 |
| Adenoma |  |  |  |  |
| Poultry |  |  |  |  |
| Boiled | 1 (Ref.) | 0.96 (0.81-1.13) | 1.00 (0.85-1.18) | 0.975 |
| Fried | 1 (Ref.) | 1.02(0.86-1.21) | 1.03 (0.87-1.23) | 0.715 |
| Marinated | 1 (Ref.) | 1.04 (0.86-1.26) | 1.05 (0.86-1.28) | 0.569 |
| Fish |  |  |  |  |
| Boiled | 1 (Ref.) | 1.18 (0.90-1.54) | 1.17 (0.89-1.55) | 0.149 |
| Fried | 1 (Ref.) | 0.93 (0.77-1.13) | 0.95 (0.78-1.17) | 0.770 |
| Egg |  |  |  |  |
| Boiled | 1 (Ref.) | 0.81 (0.67-0.99) | 0.81 (0.66-1.00) | 0.111 |
| Fried | 1 (Ref.) | 1.22 (1.04-1.43) | 1.02 (0.86-1.20) | 0.673 |
| Marinated | 1 (Ref.) | 1.11 (0.94-1.31) | 1.21 (1.03-1.43) | 0.019 |
| soy |  |  |  |  |
| Boiled | 1 (Ref.) | 1.07 (0.88-1.29) | 1.16 (0.96-1.40) | 0.120 |
| Fried | 1 (Ref.) | 1.21 (0.90-1.64) | 1.26 (0.93-1.71) | 0.216 |
| Marinated | 1 (Ref.) | 1.13 (0.91-1.40) | 1.01 (0.81-1.25) | 0.586 |

^a^ C, classification; Multivariable model adjusted for age, sex, BMI, smoking, alcohol consumption, household annual income, physical activity, vitamin supplement use, history of family colorectal cancer, regular aspirin use, educational level, food consumption of remaining cooking methods, total energy intake and healthy diet score.

**Supplementary Table 5.** Subgroup analyses for multivariable-adjusted ORs (95% CIs) of soy consumption with the prevalence of polyps or adenomas ^a^.

|  | Cases/n | Quartiles of soy consumption (g·2,000 kcal^-1^·d^-1^) | | | | P-trend | P for interaction |
| --- | --- | --- | --- | --- | --- | --- | --- |
|  |  | Q1 | Q2 | Q3 | Q4 |  |  |
| Polyp |  |  |  |  |  |  |  |
| Age |  |  |  |  |  |  | 0.344 |
| <60 years | 1,819/6,870 | 1 (Ref.) | 1.08 (0.92-1.27) | 1.21 (1.03-1.42) | 1.20 (1.02-1.41) | 0.014 |  |
| ≥60 years | 3,123/7,683 | 1 (Ref.) | 0.96 (0.84-1.09) | 1.06 (0.93-1.21) | 1.08 (0.95-1.24) | 0.115 |  |
| BMI |  |  |  |  |  |  | 0.084 |
| <24 kg/m^2^ | 2,616/8,244 | 1 (Ref.) | 1.02 (0.89-1.17) | 1.14 (0.99-1.30) | 1.22 (1.06-1.40) | 0.002 |  |
| ≥24 kg/m^2^ | 2,326/6,309 | 1 (Ref.) | 0.99 (0.85-1.16) | 1.10 (0.94-1.28) | 1.02 (0.87-1.18) | 0.566 |  |
| Sex |  |  |  |  |  |  | 0.451 |
| Men | 3,275/7,602 | 1 (Ref.) | 0.96 (0.84-1.09) | 1.08 (0.94-1.23) | 1.08 (0.94-1.23) | 0.125 |  |
| Women | 1,667/6,951 | 1 (Ref.) | 1.08 (0.92-1.27) | 1.18 (1.01-1.39) | 1.19 (1.01-1.40) | 0.020 |  |
| Physical activity |  |  |  |  |  |  | 0.420 |
| <Median | 2,658/7,276 | 1 (Ref.) | 1.01 (0.87-1.17) | 1.12 (0.98-1.30) | 1.18 (1.03-1.37) | 0.008 |  |
| ≥Median | 2,284/7,277 | 1 (Ref.) | 1.00 (0.87-1.16) | 1.12 (0.97-1.30) | 1.05 (0.90-1.21) | 0.297 |  |
| Smoking |  |  |  |  |  |  | 0.628 |
| Nonsmoker | 2,483/9,284 | 1 (Ref.) | 1.00 (0.87-1.14) | 1.15 (1.01-1.32) | 1.14 (0.99-1.30) | 0.017 |  |
| Former/current smoker | 2,459/5,269 | 1 (Ref.) | 1.02 (0.87-1.19) | 1.09 (0.93-1.27) | 1.11 (0.95-1.30) | 0.151 |  |
| Alcohol consumption |  |  |  |  |  |  | 0.359 |
| Nondrinker | 2,551/8,567 | 1 (Ref.) | 1.07 (0.94-1.23) | 1.10 (0.96-1.26) | 1.13 (0.99-1.30) | 0.074 |  |
| Drinker | 2,391/5,986 | 1 (Ref.) | 0.94 (0.80-1.09) | 1.16 (0.99-1.35) | 1.12 (0.96-1.31) | 0.031 |  |
| Educational level |  |  |  |  |  |  | 0.228 |
| < middle school | 4,228/12,440 | 1 (Ref.) | 0.99 (0.88-1.10) | 1.15 (1.03-1.28) | 1.13 (1.01-1.26) | 0.005 |  |
| ≥ middle school | 714/2,113 | 1 (Ref.) | 1.10 (0.81-1.49) | 0.96 (0.71-1.29) | 1.08 (0.80-1.45) | 0.864 |  |
| Total energy intake |  |  |  |  |  |  | 0.877 |
| <Median | 2,085/7,276 | 1 (Ref.) | 1.13 (0.98-1.32) | 1.18 (1.01-1.37) | 1.16 (1.00-1.35) | 0.048 |  |
| ≥Median | 2,857/7,277 | 1 (Ref.) | 0.90 (0.78-1.04) | 1.08 (0.94-1.24) | 1.09 (0.95-1.25) | 0.062 |  |
| Healthy diet score |  |  |  |  |  |  | 0.151 |
| <Median | 2,147/5,811 | 1 (Ref.) | 0.90 (0.77-1.06) | 1.06 (0.91-1.24) | 0.97 (0.83-1.14) | 0.749 |  |
| ≥Median | 2,795/8,742 | 1 (Ref.) | 1.08 (0.94-1.24) | 1.17 (1.02-1.34) | 1.23 (1.07-1.40) | 0.002 |  |
| Adenoma |  |  |  |  |  |  |  |
| Age |  |  |  |  |  |  | 0.843 |
| <60 years | 936/6,870 | 1 (Ref.) | 1.08 (0.88-1.32) | 1.26 (1.03-1.54) | 1.25 (1.01-1.53) | 0.015 |  |
| ≥60 years | 1,742/7,683 | 1 (Ref.) | 0.92 (0.78-1.08) | 1.06 (0.91-1.23) | 1.13 (0.97-1.32) | 0.044 |  |
| BMI |  |  |  |  |  |  | 0.455 |
| <24 kg/m^2^ | 1,455/8,244 | 1 (Ref.) | 0.95 (0.80-1.12) | 1.11 (0.94-1.31) | 1.21 (1.03-1.43) | 0.007 |  |
| ≥24 kg/m^2^ | 1,223/6,309 | 1 (Ref.) | 1.01 (0.84-1.22) | 1.14 (0.95-1.37) | 1.12 (0.94-1.35) | 0.116 |  |
| Sex |  |  |  |  |  |  | 0.354 |
| Men | 1,818/7,602 | 1 (Ref.) | 1.00 (1.12-0.96) | 1.12 (0.96-1.30) | 1.23 (1.05-1.43) | 0.003 |  |
| Women | 860/6,951 | 1 (Ref.) | 1.00 (1.15-0.93) | 1.15 (0.93-1.41) | 1.07 (0.87-1.32) | 0.310 |  |
| Physical activity |  |  |  |  |  |  | 0.815 |
| <Median | 1,480/7,276 | 1 (Ref.) | 0.97 (0.81-1.16) | 1.13 (0.96-1.34) | 1.18 (1.00-1.40) | 0.014 |  |
| ≥Median | 1,198/7,277 | 1 (Ref.) | 0.98 (0.82-1.17) | 1.12 (0.93-1.34) | 1.14 (0.95-1.36) | 0.085 |  |
| Smoking |  |  |  |  |  |  | 0.532 |
| Nonsmoker | 1,317/9,284 | 1 (Ref.) | 0.99 (0.83-1.17) | 1.15 (0.98-1.36) | 1.13 (0.95-1.34) | 0.059 |  |
| Former/current smoker | 1,361/5,269 | 1 (Ref.) | 0.96 (0.80-1.15) | 1.10 (0.92-1.31) | 1.22 (1.02-1.46) | 0.011 |  |
| Alcohol consumption |  |  |  |  |  |  | 0.066 |
| Nondrinker | 1,351/8,567 | 1 (Ref.) | 0.92 (0.78-1.10) | 1.05 (0.89-1.24) | 1.07 (0.91-1.27) | 0.219 |  |
| Drinker | 1,327/5,986 | 1 (Ref.) | 1.04 (0.87-1.25) | 1.22 (1.02-1.46) | 1.30 (1.09-1.56) | 0.001 |  |
| Educational level |  |  |  |  |  |  | 0.081 |
| < middle school | 2,294/12,440 | 1 (Ref.) | 0.95 (0.83-1.08) | 1.15 (1.01-1.31) | 1.19 (1.05-1.36) | 0.001 |  |
| ≥ middle school | 384/2,113 | 1 (Ref.) | 1.12 (0.77-1.61) | 0.97 (0.68-1.39) | 1.01 (0.71-1.44) | 0.782 |  |
| Total energy intake |  |  |  |  |  |  | 0.359 |
| <Median | 1,083/7,276 | 1 (Ref.) | 0.99 (0.82-1.19) | 1.17 (0.97-1.41) | 1.10 (0.91-1.33) | 0.141 |  |
| ≥Median | 1,595/7,277 | 1 (Ref.) | 0.96 (0.81-1.14) | 1.09 (0.92-1.28) | 1.22 (1.03-1.43) | 0.007 |  |
| Healthy diet score |  |  |  |  |  |  | 0.419 |
| <Median | 1,197/5,811 | 1 (Ref.) | 0.90 (0.75-1.08) | 1.04 (0.87-1.25) | 1.08 (0.90-1.30) | 0.235 |  |
| ≥Median | 1,481/8,742 | 1 (Ref.) | 1.04 (0.88-1.23) | 1.20 (1.01-1.41) | 1.25 (1.06-1.47) | 0.003 |  |

^a^ Q, quartile; Multivariable model adjusted for age, sex, BMI, smoking, alcohol consumption, household annual income, physical activity, vitamin supplement use, history of family colorectal cancer, regular aspirin use, educational level, total energy intake and healthy diet score.

**Supplementary Table 6.** Subgroup analyses for multivariable-adjusted ORs (95% CIs) of fried soy consumption with the prevalence of polyps or adenomas ^a^.

|  | Cases/n | Classifications of fried soy consumption (g·2,000 kcal^-1^·d^-1^) | | | P-trend | P for interaction |
| --- | --- | --- | --- | --- | --- | --- |
|  |  | C1 | C2 | C3 |  |  |
| Polyp |  |  |  |  |  |  |
| Age |  |  |  |  |  | 0.960 |
| <60 years | 720/2,530 | 1 (Ref.) | 1.02 (0.62-1.68) | 1.10 (0.67-1.82) | 0.433 |  |
| ≥60 years | 1,447/3,376 | 1 (Ref.) | 1.15 (0.85-1.55) | 1.35 (1.00-1.83) | 0.010 |  |
| BMI |  |  |  |  |  | 0.142 |
| <24 kg/m^2^ | 1,087/3,146 | 1 (Ref.) | 1.10 (0.77-1.58) | 1.38 (0.96-1.99) | 0.004 |  |
| ≥24 kg/m^2^ | 1,080/2,760 | 1 (Ref.) | 1.19 (0.82-1.73) | 1.20 (0.82-1.75) | 0.560 |  |
| Sex |  |  |  |  |  | 0.947 |
| Men | 1,427/3,150 | 1 (Ref.) | 1.25 (0.89-1.76) | 1.39 (0.98-1.96) | 0.048 |  |
| Women | 740/2,756 | 1 (Ref.) | 0.99 (0.67-1.47) | 1.13 (0.76-1.68) | 0.195 |  |
| Physical activity |  |  |  |  |  | 0.553 |
| <Median | 1,190/2,958 | 1 (Ref.) | 1.16 (0.83-1.64) | 1.35 (0.96-1.90) | 0.029 |  |
| ≥Median | 977/2,948 | 1 (Ref.) | 1.08 (0.72-1.61) | 1.18 (0.79-1.77) | 0.235 |  |
| Smoking |  |  |  |  |  | 0.776 |
| Nonsmoker | 1,037/3,632 | 1 (Ref.) | 1.14 (0.81-1.62) | 1.30 (0.92-1.84) | 0.054 |  |
| Former/current smoker | 1,130/2,274 | 1 (Ref.) | 1.14 (0.77-1.69) | 1.29 (0.87-1.92) | 0.096 |  |
| Alcohol consumption |  |  |  |  |  | 0.199 |
| Nondrinker | 1,185/3,746 | 1 (Ref.) | 0.97 (0.72-1.32) | 1.11 (0.81-1.51) | 0.148 |  |
| Drinker | 982/2,160 | 1 (Ref.) | 1.73 (1.07-2.79) | 1.96 (1.21-3.18) | 0.014 |  |
| Educational level |  |  |  |  |  | 0.673 |
| < middle school | 1,862/5,018 | 1 (Ref.) | 1.13 (0.86-1.47) | 1.28 (0.97-1.67) | 0.020 |  |
| ≥ middle school | 305/888 | 1 (Ref.) | 1.13 (0.45-2.86) | 1.33 (0.53-3.32) | 0.292 |  |
| Total energy intake |  |  |  |  |  | 0.523 |
| <Median | 920/2,953 | 1 (Ref.) | 0.93 (0.66-1.29) | 1.10 (0.78-1.54) | 0.127 |  |
| ≥Median | 1,247/2,953 | 1 (Ref.) | 1.48 (0.98-2.24) | 1.59 (1.05-2.41) | 0.067 |  |
| Healthy diets core |  |  |  |  |  | 0.724 |
| <Median | 1,059/2,683 | 1 (Ref.) | 1.18 (0.81-1.71) | 1.27 (0.87-1.85) | 0.191 |  |
| ≥Median | 1,108/3,223 | 1 (Ref.) | 1.11 (0.77-1.59) | 1.30 (0.91-1.87) | 0.028 |  |
| Adenoma |  |  |  |  |  |  |
| Age |  |  |  |  |  | 0.150 |
| <60 years | 400/2,530 | 1 (Ref.) | 1.01 (0.55-1.84) | 0.93 (0.50-1.71) | 0.536 |  |
| ≥60 years | 894/3,376 | 1 (Ref.) | 1.23 (0.87-1.74) | 1.37 (0.96-1.94) | 0.060 |  |
| BMI |  |  |  |  |  | 0.064 |
| <24 kg/m^2^ | 670/3,146 | 1 (Ref.) | 1.59 (1.01-2.49) | 1.80 (1.14-2.83) | 0.017 |  |
| ≥24 kg/m^2^ | 624/2,760 | 1 (Ref.) | 0.97 (0.64-1.48) | 0.92 (0.60-1.40) | 0.530 |  |
| Sex |  |  |  |  |  | 0.235 |
| Men | 855/3,150 | 1 (Ref.) | 1.27 (0.85-1.88) | 1.38 (0.92-2.05) | 0.090 |  |
| Women | 439/2,756 | 1 (Ref.) | 1.11 (0.69-1.79) | 1.07 (0.66-1.72) | 0.746 |  |
| Physical activity |  |  |  |  |  | 0.295 |
| <Median | 711/2,958 | 1 (Ref.) | 1.30 (0.87-1.94) | 1.38 (0.92-2.07) | 0.154 |  |
| ≥Median | 583/2,948 | 1 (Ref.) | 1.08 (0.68-1.73) | 1.08 (0.67-1.74) | 0.843 |  |
| Smoking |  |  |  |  |  | 0.270 |
| Nonsmoker | 616/3,632 | 1 (Ref.) | 1.27 (0.84-1.93) | 1.24 (0.81-1.89) | 0.679 |  |
| Former/current smoker | 678/2,274 | 1 (Ref.) | 1.11 (0.71-1.73) | 1.25 (0.80-1.96) | 0.146 |  |
| Alcohol consumption |  |  |  |  |  | 0.234 |
| Nondrinker | 697/3,746 | 1 (Ref.) | 1.02 (0.71-1.47) | 1.06 (0.74-1.52) | 0.683 |  |
| Drinker | 597/2,160 | 1 (Ref.) | 1.90 (1.06-3.41) | 1.99 (1.11-3.58) | 0.118 |  |
| Educational level |  |  |  |  |  | 0.089 |
| < middle school | 1,126/5,018 | 1 (Ref.) | 1.23 (0.89-1.69) | 1.32 (0.96-1.82) | 0.099 |  |
| ≥ middle school | 168/888 | 1 (Ref.) | 0.85 (0.31-2.32) | 0.75 (0.28-2.03) | 0.435 |  |
| Total energy intake |  |  |  |  |  | 0.555 |
| <Median | 539/2,953 | 1 (Ref.) | 1.00 (0.68-1.48) | 1.06 (0.71-1.58) | 0.602 |  |
| ≥Median | 755/2,953 | 1 (Ref.) | 1.58 (0.97-2.57) | 1.61 (0.98-2.62) | 0.242 |  |
| Healthy diets core |  |  |  |  |  | 0.230 |
| <Median | 644/2,683 | 1 (Ref.) | 1.20 (0.78-1.84) | 1.37 (0.89-2.11) | 0.082 |  |
| ≥Median | 650/3,223 | 1 (Ref.) | 1.26 (0.81-1.94) | 1.18 (0.76-1.82) | 0.982 |  |

^a^ C, classification; Multivariable model adjusted for age, sex, BMI, smoking, alcohol consumption, household annual income, physical activity, vitamin supplement use, history of family colorectal cancer, regular aspirin use, educational level, total energy intake and healthy diet score.

**Supplementary Table 7.** Sensitivity analyses for the multivariable-adjusted ORs (95% CIs) of colorectal polyp or adenoma prevalence according to soy consumption ^a^.

|  | Cases/n | Quartiles of bean consumption (g·2,000 kcal^-1^·d^-1^) | | | | |
| --- | --- | --- | --- | --- | --- | --- |
|  |  | Q1 | Q2 | Q3 | Q4 | P-trend |
| Polyp |  |  |  |  |  |  |
| Excluding individuals with extreme energy intake | 4,769/14,172 | 1 (Ref.) | 1.00 (0.90-1.11) | 1.12 (1.01-1.25) | 1.12 (1.01-1.25) | 0.007 |
| Excluding individuals with extreme BMI | 4,936/14,537 | 1 (Ref.) | 1.00 (0.91-1.11) | 1.12 (1.01-1.24) | 1.12 (1.01-1.24) | 0.009 |
| Further adjust for calcium supplement use | 4,942/14,553 | 1 (Ref.) | 1.00 (0.91-1.11) | 1.12 (1.01-1.24) | 1.12 (1.01-1.24) | 0.007 |
| Further adjust for baseline diabetes | 4,942/14,553 | 1 (Ref.) | 1.00 (0.91-1.11) | 1.12 (1.01-1.24) | 1.12 (1.01-1.24) | 0.007 |
| Adenoma |  |  |  |  |  |  |
| Excluding individuals with extreme energy intake | 2,588/14,172 | 1 (Ref.) | 0.95 (0.84-1.08) | 1.12 (0.99-1.27) | 1.15 (1.02-1.31) | 0.004 |
| Excluding individuals with extreme BMI | 2,674/14,537 | 1 (Ref.) | 0.97 (0.86-1.10) | 1.12 (0.99-1.27) | 1.16 (1.03-1.31) | 0.003 |
| Further adjust for calcium supplement use | 2,678/14,553 | 1 (Ref.) | 0.97 (0.86-1.10) | 1.12 (0.99-1.26) | 1.16 (1.03-1.31) | 0.003 |
| Further adjust for baseline diabetes | 2,678/14,553 | 1 (Ref.) | 0.97 (0.86-1.10) | 1.12 (0.99-1.27) | 1.17 (1.03-1.32) | 0.002 |

^a^ Q, quartile; Multivariable model adjusted for age, sex, BMI, smoking, alcohol consumption, household annual income, physical activity, vitamin supplement use, history of family colorectal cancer, regular aspirin use, educational level, total energy intake and healthy diet score.

**Supplementary Table 7.** Sensitivity analyses for the multivariable-adjusted ORs (95% CIs) of colorectal polyp or adenoma prevalence according to soy consumption in different cooking methods ^a^.

|  | Cases/n | Classification of soy consumption (g·2,000 kcal^-1^·d^-1^) | | | |
| --- | --- | --- | --- | --- | --- |
|  |  | C1 | C2 | C3 | P-trend |
| Boiled soys |  |  |  |  |  |
| Polyp |  |  |  |  |  |
| Excluding individuals with extreme energy intake | 2,058/5,672 | 1 (Ref.) | 1.24 (1.03-1.49) | 0.95 (0.77-1.18) | 0.912 |
| Excluding individuals with extreme BMI | 2,164/5,902 | 1 (Ref.) | 1.01 (0.85-1.20) | 0.99 (0.84-1.17) | 0.946 |
| Further adjusting for calcium supplement use | 2,167/5,906 | 1 (Ref.) | 1.00 (0.84-1.18) | 0.99 (0.84-1.18) | 0.930 |
| Further adjusting for baseline diabetes | 2,167/5,906 | 1 (Ref.) | 1.00 (0.84-1.18) | 0.99 (0.84-1.18) | 0.921 |
| Other boiled food intake ^b^ | 2,167/5,906 | 1 (Ref.) | 1.04 (0.87-1.23) | 0.99 (0.84-1.17) | 0.948 |
| Adenoma |  |  |  |  |  |
| Excluding individuals with extreme energy intake | 1,231/5,672 | 1 (Ref.) | 1.07 (0.88-1.31) | 1.21 (1.00-1.47) | 0.056 |
| Excluding individuals with extreme BMI | 1,291/5,902 | 1 (Ref.) | 1.06 (0.87-1.29) | 1.16 (0.96-1.39) | 0.126 |
| Further adjust for calcium supplement use | 1,294/5,906 | 1 (Ref.) | 1.07 (0.88-1.30) | 1.16 (0.96-1.40) | 0.114 |
| Further adjust for baseline diabetes | 1,294/5,906 | 1 (Ref.) | 1.07 (0.88-1.29) | 1.16 (0.96-1.40) | 0.123 |
| Other boiled food intake ^b^ | 1,294/5,906 | 1 (Ref.) | 1.09 (0.90-1.32) | 1.17 (0.97-1.41) | 0.076 |
| Fried soys |  |  |  |  |  |
| Polyp |  |  |  |  |  |
| Excluding individuals with extreme energy intake | 2,058/5,672 | 1 (Ref.) | 1.17 (0.90-1.52) | 1.30 (0.99-1.69) | 0.025 |
| Excluding individuals with extreme BMI | 2,164/5,902 | 1 (Ref.) | 1.14 (0.88-1.47) | 1.25 (0.96-1.62) | 0.040 |
| Further adjusting for calcium supplement use | 2,167/5,906 | 1 (Ref.) | 1.13 (0.88-1.47) | 1.28 (0.99-1.66) | 0.012 |
| Further adjusting for baseline diabetes | 2,167/5,906 | 1 (Ref.) | 1.13 (0.87-1.46) | 1.28 (0.99-1.66) | 0.014 |
| Other fried food intake ^c^ | 2,167/5,906 | 1 (Ref.) | 1.15 (0.88-1.49) | 1.27 (0.98-1.65) | 0.026 |
| Adenoma |  |  |  |  |  |
| Excluding individuals with extreme energy intake | 1,231/5,672 | 1 (Ref.) | 1.20 (0.88-1.63) | 1.28 (0.94-1.75) | 0.129 |
| Excluding individuals with extreme BMI | 1,291/5,902 | 1 (Ref.) | 1.19 (0.88-1.61) | 1.27 (0.93-1.72) | 0.132 |
| Further adjusting for calcium supplement use | 1,294/5,906 | 1 (Ref.) | 1.22 (0.90-1.65) | 1.26 (0.93-1.71) | 0.211 |
| Further adjusting for baseline diabetes | 1,294/5,906 | 1 (Ref.) | 1.21 (0.90-1.64) | 1.26 (0.93-1.71) | 0.204 |
| Other fried food intake ^c^ | 1,294/5,906 | 1 (Ref.) | 1.22 (0.90-1.66) | 1.32 (0.97-1.80) | 0.061 |
| Marinated soys |  |  |  |  |  |
| Polyp |  |  |  |  |  |
| Excluding individuals with extreme energy intake | 2,058/5,672 | 1 (Ref.) | 0.98 (0.82-1.17) | 1.02 (0.86-1.21) | 0.581 |
| Excluding individuals with extreme BMI | 2,164/5,902 | 1 (Ref.) | 1.18 (0.99-1.42) | 0.98 (0.80-1.20) | 0.591 |
| Further adjusting for calcium supplement use | 2,167/5,906 | 1 (Ref.) | 1.23 (1.02-1.49) | 0.98 (0.81-1.19) | 0.582 |
| Further adjusting for baseline diabetes | 2,167/5,906 | 1 (Ref.) | 1.23 (1.02-1.49) | 0.98 (0.80-1.18) | 0.593 |
| Other marinated food intake ^d^ | 2,167/5,906 | 1 (Ref.) | 1.18 (0.98-1.41) | 0.98 (0.79-1.20) | 0.561 |
| Adenoma |  |  |  |  |  |
| Excluding individuals with extreme energy intake | 1,231/5,672 | 1 (Ref.) | 1.16 (0.94-1.43) | 0.96 (0.75-1.23) | 0.781 |
| Excluding individuals with extreme BMI | 1,291/5,902 | 1 (Ref.) | 1.13 (0.92-1.38) | 0.96 (0.76-1.22) | 0.860 |
| Further adjusting for calcium supplement use | 1,294/5,906 | 1 (Ref.) | 1.13 (0.91-1.40) | 1.01 (0.81-1.26) | 0.647 |
| Further adjusting for baseline diabetes | 1,294/5,906 | 1 (Ref.) | 1.12 (0.91-1.39) | 1.01 (0.81-1.26) | 0.651 |
| Other marinated food intake ^d^ | 2,167/5,906 | 1 (Ref.) | 1.13 (0.92-1.39) | 0.98 (0.77-1.23) | 0.720 |

^a^ C, classification; Multivariable model adjusted for age, sex, BMI, smoking, alcohol consumption, household annual income, physical activity, vitamin supplement use, history of family colorectal cancer, regular aspirin use, educational level, food consumption of remaining cooking methods, total energy intake and healthy diet score.

^b^ Multivariable model adjusted for age, sex, BMI, smoking, alcohol consumption, household annual income, physical activity, vitamin supplement use, history of family colorectal cancer, regular aspirin use, educational level, food consumption of remaining cooking methods, total energy intake, cooking diet score (calculated excluding fish), other boiled food intake, all fried food and all marinated food.

^c^ Multivariable model adjusted for age, sex, BMI, smoking, alcohol consumption, household annual income, physical activity, vitamin supplement use, history of family colorectal cancer, regular aspirin use, educational level, food consumption of remaining cooking methods, total energy intake, cooking diet score (calculated excluding fish), other fried food intake, all boiled food and all marinated food.

^d^ Multivariable model adjusted for age, sex, BMI, smoking, alcohol consumption, household annual income, physical activity, vitamin supplement use, history of family colorectal cancer, regular aspirin use, educational level, food consumption of remaining cooking methods, total energy intake, cooking diet score (calculated excluding fish), other marinated food intake, all boiled food and all fried food.
